# Prevalence and risk factors of neurological impairment among children aged 6–9 years: from population based cross sectional study in western Kenya

**DOI:** 10.1186/1471-2431-12-186

**Published:** 2012-12-03

**Authors:** Yoshito Kawakatsu, Satoshi Kaneko, Mohamed Karama, Sumihisa Honda

**Affiliations:** 1 JICA SEMAH project, Kisumu, Kenya; 2 Graduate school of Biomedical science, Nagasaki University, Nagasaki, Japan; 3 Department of EcoEpidemiology, Institute of Tropical Medicine, Nagasaki University, Nagasaki, Japan; 4 NUITM-KEMRI project, Nairobi, Kenya; 5 Centre of Public Health Research, Kenya Medical Research Institute (KEMRI), Nairobi, Kenya; 6 , Postal Address: 852-8523 1-12-4, Sakamoto, Nagasaki-city, Nagasaki, Japan

**Keywords:** Disability, Neuroepidemiology, Pediatric, Kenya, Risk factor

## Abstract

**Background:**

The burden of disability is more severe among children in low income countries. Moreover, the number of children with disabilities (CWDs) in sub-Saharan Africa is predicted to increase with reduction in child mortality. Although the issue on CWDs is important in sub-Saharan Africa, there are few researches on risk factors of disabilities. The purpose of this study was to evaluate the risk factors of neurological impairment (NI) among children in western Kenya.

**Methods:**

The present study was conducted in Mbita district (which has high HIV infectious prevalence), Kenya from April 2009 to December 2010. The study consisted of two phases. In phase 1, the Ten Question Questionnaire (TQQ) was administered to all 6362 caregivers of children aged 6–9 years. In phase two, all 413 children with TQQ positive and a similar number of controls (n=420) which were randomly selected from children with TQQ negative were examined for physical and cognitive status. In addition, a structured questionnaire was also conducted to their caregivers.

**Results:**

The prevalence was estimated to be 29/1000. Among the types of impairments, cognitive impairment was the most common (24/1000), followed by physical impairment (5/1000). In multivariate analysis, having more than five children [adjusted odds ratio (AOR): 2.85; 95%IC: 1.25 – 6.49; p=0.013], maternal age older than 35 years old [AOR: 2.31; 95%IC: 1.05 – 5.07; p=0.036] were significant factors associated with NI. In addition, monthly income under 3000 ksh [AOR: 2.79; 95%IC: 1.28 – 6.08; p=0.010] and no maternal tetanus shot during antenatal care [AOR: 5.17; 95%IC: 1.56 – 17.14; p=0.007] were also significantly related with having moderate/severe neurological impairment.

**Conclusion:**

It was indicated that increasing coverage of antenatal care including maternal tetanus shot and education of how to take care of neonatal children to prevent neurological impairment are important.

## Background

The average global prevalence of moderate and severe disability is estimated to be 5% in children aged 0–14 years. Disability among children in low-income countries is more common than high-income countries [1]. The number of children with disabilities (CWDs) in Sub-Sahara Africa is estimated to increase with the reduction in child mortality [2]. Furthermore, neurological impairment such as epilepsy, hearing impairment, vision impairment, physical impairment and cognitive impairments is one of the important causes of disability and death. It estimated to account for >28% of years lived with disability and to be responsible for at least one in every nine deaths [3].

Main risk factors of severe and moderate disabilities in low-income countries were generally considered genetic factors [4,5], nutritional deficiencies [6-9], infections [10], prenatal and neonatal factors [11,12] and socio-economic factors [13,14]. However, most of the studies focused on one impairment/disability [15-18] and some researches didn’t conduct physical assessment to diagnose child’s disability [9,19]. In addition, there are few researches on causes and risk factors of NI in sub-Saharan Africa, especially high prevalence area of malaria and HIV infection.

This research aimed to assess prevalence of NI and evaluate risk factors of NI among children aged 6–9 years by conducting structured questionnaire and physical assessments at the area of high malaria and HIV prevalence in Kenya.

## Methods

### Research Site

This study was conducted in Gembe West, Gembe East, Rusinga West and Rusinga East, Mbita district, Nyanza province, Kenya, located on the lakeside of Lake Victoria. This is one of the poorest areas in Kenya [20] and the residents are primarily, subsistence farmers or fishermen. Moreover, this area has one of the highest prevalence rates of malaria and HIV infection [21]. Health and Demographic Surveillance System (HDSS) project in this area is being conducted by Kenya Medical Research Institute (KEMRI) - Nagasaki University Institute of Tropical Medicine (NUITM) project [22].

### Research population

The research population consisted of the all 6263 children, aged 6–9 years, and their caregivers in research site. Their main tribe is the Luo tribe and their main languages are Luo language, followed by Swahili and English. The age group of 6–9 years was selected because of difficulties in identifying impairments in children younger than 6 years old and the lack of cross-cultural assessment tools for cognitive impairment in young children.

In phase one, we targeted all 6263 caregivers of children aged 6–9 years in the research area. The sample size for phase two was calculated according to L.Naing [23]. With estimated prevalence of CWDs in Kenya (10%), confidence level of 95% and with a relative precision of 2.5% points on each side, a sample of 813 children was needed.

### Study design and data collection

**Figure 1:**
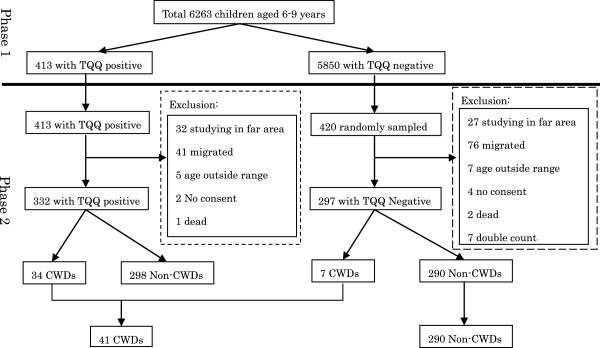
**Study procedure.** In phase one, we performed TQQ to all 6263 caregivers who has children aged 6-9 years. There were 413 children with TQQ positive and 5850 with negative. In phase two, all children with positive response (413) and a similar number of children with negative (420) were conducted physical, neurological and cognitive assessments and their caregivers were administered structured questionnaire. Finally, 41 children with disabilities were identified.

This study was population based cross-sectional survey conducted from April 2009 to November 2010. There were two phases in this research. In phase one, the Ten Question Questionnaire (TQQ) was administered to all 6263 caregivers of children aged 6–9 years in April 2009 (Figure 1). In phase two, all children (413) with at least one positive response in TQQ and a similar number of children (420), randomly selected from those who had all negative response in TQQ were selected. Total 833 children were examined using the physical, neurological and cognitive assessments. A structured questionnaire on socio-demographic characteristics and potential risk factors for NI was also conducted to their caregivers in November 2010. In this study, birth difficulty was defined as any difficulties such as heavy bleeding, strange breech positioning, or asphyxia at the birth. Neonatal insult was defined by a positive history of tetanus, jaundice, sepsis or any other severe infection during the neonatal period.

### Instruments

#### Ten Questions Questionnaire (TQQ)

The Ten Questions Questionnaire (TQQ) developed by WHO is a convenient questionnaire focusing on the child’s functional abilities and is used to detect NI among children aged 2–9 years in community settings [24]. TQQ has been used widely to screen for childhood impairment in low- and middle-income counties [9,12,14]. The validity of the TQQ has been reported in some countries such as Bangladesh [25], Jamaica [25], Pakistan [25], India [26] and Kenya [27]. We conducted one day training to research assistants to improve their understanding on this question. Although most of the participants could understand English in this area, the questionnaire was translated into Luo language in case that they could not understand English.

### Physical, neurological and cognitive assessments

The word of NI mentioned in this research belongs to body functions & structures part in the International Classification of Functioning, Disability, and Health (ICF) and contained cognitive impairment, physical impairment, epilepsy, hearing impairment and visual impairment. The assessments to diagnose NI included physical examination [28] including measurement of height and weight to assess motor impairment and a vision test (Landolt Chart) [29]. Hearing test by three screening tests (Behavioral Observation Audiometry (BOA): bell-tone, paper crash test, small voice) was also performed. Almost quarter subjects in phase 2 were re-checked by using an audiometer [30] (KS8: PC Werth Ltd) . In addition, the diagnosis of epilepsy was based on the history gathered from their caregivers about the child’s epilepsy-like symptoms.

Cross-cultural cognitive assessment was also used [31]. This cognitive assessment included seven batteries which tested verbal and non-verbal skills, namely, “Digit span (phonological loop component of working memory)”, “Corsi Block ( operational skill of the visuospatial sketch-pad component of working memory)”, “Verbal fluency (retrieval function from long term memory)”, “Silly sentence (general intelligence and speed of access to semantic memory)”,”Visual search (speed of visual information processing)”, “Free recall (Long term memory) [32]”, and ”Vocabulary learning (prose learning) [31]”. These seven cognitive assessments were performed for the target children. These seven cognitive assessments were analyzed by using factor analysis and summarized as factor 1 and 2. Factor one significantly correlated with Digit span, Visual search, Silly sentence, and Free recall, while factor two significantly correlated with Corsi block, Verbal fluency. Finally, all suspected cases by using above assessments were re-evaluated by a physical therapist.

Severe cognitive impairment was defined as the inability to successfully perform even one of the cognitive assessment batteries. Moderate cognitive impairment was defined on the basis of the two factor’s scores. The cutoff point was established as less than 1% of each factor scores in 296 children with negative response of TQQ. Since the factor scores were not associated with age and sex, age- or sex-specific cutoff points were not determined in the present study. For other domains, the definition of severity was shown in Table 1.

**Table 1:** Definitions of moderate and severe impairment

**Impairment**	**Moderate**	**Severe**
Cognitive	Under 1% of factor 1 OR factor 2 in 296 children with negative response of TQQ	Inability to successfully complete even one cognitive test item
Motor ^a^	Difficulty in grip, dressing and sitting. Ability to move around with help.	Inability to walk and absence of functional use of hands
Epilepsy ^a^	More than one non-febrile seizure per month	More than one non-febrile seizure per week.
Hearing ^a^	At least one positive response in screening tests and 41–70 dB loss in the best ear.	More than 70 dB loss in the best ear
Vision ^a^	Visual acuity less than 6/18 or better than 6/60	Visual acuity worse than 6/60 meters or better than 3/60

^a^ Adopted from WHO procedure manual.

### Data storage and analysis

Data from all phases were double-entered after the verification of the data had been performed and stored using Epi info version 3.5. Statistical analysis was performed by using STATA version 10 (STATA Corporation, TX, USA).

Factor analysis was used to refine factor structure in seven cognitive assessments. Thirteen variables were considered as the potential risk factors. The potential risk factors were dichotomized and coded. Moreover, these potential risk factors in children aged 6–9 years were analyzed using univariate analysis and linear logistic models with 13 potential factors as covariates. Starting with a logistic model including all of these covariates, we selected the most appropriate model on the basis of Akaike’s information criterion (AIC) [33]. Once the most appropriate model was selected, maximum likelihood estimation of the model parameters was conducted and then the odds ratio and the 95% confidence interval were calculated for each covariate in the model.

### Ethical approval

Informed consents from all guardians of target children were obtained after fully explanation of the study purpose and possible consequences. This study was approved by the Ethical Review Committee of Kenya Medical Research Institute (KEMRI SSC No. 1088) and National Council for Science and Technology in Kenya (Approval number: NCST/RR1/12/1/SS/150/5). In addition, the ethical committee of the Institute of Tropical Medicine, Nagasaki University (Approval number: 06060604) and the ethics committee of International Health Development, Graduate School of Nagasaki University (Approval number: 0012) were approved this study.

## Results

All 6263 children aged 6–9 years were screened in phase one. Among the screened children, 50.4% of them were male. Moreover, 26.8% were 6 years old (72–84 months), 22.6% were 7 years old (84–96 months), 25.9% were 8 years old (96–108 month), and 24.8% were 9 years old (108–120 months). Through the TQQ in phase one, 413 (6.5%) had at least one positive response on TQQ. The assessments to TQQ positive group in phase two could not be conducted for 81 children for different reasons (32 children left the study, 41 migrated, 5 under or over the age based on phase-2 results, 2 refused for consent, and 1 died). Finally, 332 (80%) out of 413 children with at least one positive response on TQQ were assessed during phase two. Similarly, 420 children out of 5850 who had a negative response on TQQ were selected using simple random-sampling. The assessments to TQQ negative group in phase two could not be conducted for 123 children for different reasons (26 children left the study, 77 migrated, 4 under or over the age based on phase-2 results, 7 refused for consent, 2 died and 7 double counted). Finally, 297 (70%) of the 420 children without a positive response were assessed.

Forty-one children were diagnosed as having NI after phase two assessments, 34 of them were TQQ positive group and 7 of them were in TQQ negative group. Estimated prevalence of moderate/severe NI was 29/1000. There were no significant differences in prevalence between boys and girls or age groups. Table 2 presents the prevalence of total and specific impairment. The prevalence for specific impairments varied widely. Among specific impairments, cognitive impairment was the most common at 24/1000, followed by motor impairment with the estimated prevalence of 5/1000. Epilepsy, hearing and visual impairments were rare in this study with the estimated prevalence of less than 1/1000.

**Table 2:** Estimated prevalence* of moderate/severe impairment in Mbita district, [ per 1000 children (95%CI)]

**Impairment**	**All children**	**Boys**	**Girls**
	**(95% CI)**	**(95% CI)**	**(95% CI)**
Any impairment	29.3 (12.99-45.61)	38.4 (1.80-74.94)	18.3 (4.25-56.47)
Cognitive	24.0 (8.91-39.13)	29.7 (9.71-152.94)	16.6 (3.07-54.24)
Epilepsy	0.64 (0.13-13.50)	1.1 (0.23-25.51)	0 (0.00-25.83)
Hearing	0.8 (0.23-13.81)	0.7 (0.09-24.94)	0.9 (0.10-27.34)
Motor	4.9 (0.81-20.92)	7.5 (0.56-37.41)	1.7 (0.47-28.63)
Visual	0.2 (0.00-12.82)	0.4 (0.00-24.32)	0 (0.00-25.83)
Total population	6363	3159	3104

*Formula to calculation of estimated prevalence = [(all TQQ positive × CWDs with TQQ positive/observed subjects with TQQ positive)+(all TQQ negative× CWDs with TQQ negative / observed subjects with TQQ negative)]/ 6263×1000.

Some demographic characteristics and factors considered as potential risk factors for NI are shown in Table 3. Compared to 290 children with TQQ negative, CWDs were significantly less likely to receive antenatal care (P=0.003), to get a maternal tetanus shot during antenatal care (P=0.004) and to receive full vaccination (P=0.01). In addition, maternal age higher than 35 years old (adjusted adds ratio (AOR): 2.31; 95%IC: 1.05 – 5.07; p=0.036), having more than 5 own children (AOR: 2.85; 95%IC: 1.25 – 6.49; p=0.013), monthly income under 3000 ksh (AOR: 2.79; 95%IC: 1.28 – 6.08; p=0.010) and no maternal tetanus shot in antenatal care (AOR: 5.17; 95%IC: 1.56 – 17.14; p=0.007) were found to have significant association with moderate/severe NI by using multivariate analysis (Table 4).

**Table 3:** Univariate analysis of risk factors for neurological impairment

**Variables**	**Moderate/severe impairment**		**crude OR**^**a**^	**95%CI**
	**CWDs (N=41) N (%)**	**TQQ negative(N=290) N (%)**		
**Maternal age**				
< 32 years old	19 (46.3)	168 (57.9)	Ref.	
≥ 32 years old	22 (53.7)	122 (42.1)	1.594	0.827 - 3.075
**Education level**				
Primary level or more	18 (43.9)	143 (49.3)	Ref.	
No formal education	23 (56.1)	147 (50.7)	1.243	0.644 – 2.401
**ANC visit**				
Less than 2 times	32 (78.0)	268 (92.4)	Ref.	
More than 3 times	9 (22.0)	22 (7.6)	3.426^**^	1.453 – 8.079
**Tetanus shot**				
Tetanus shot in antenatal care	30 (85.7)	268 (92.4)	Ref.	
No tetanus shot	5 (14.3)	22 (7.6)	4.685^**^	1.473 – 14.89
**Sex of children**				
Male	27 (65.9)	149 (51.4)	Ref.	
Female	14 (34.1)	141 (48.6)	1.825	0.920 – 3.621
**Income level**				
Over 3000 ksh per month	25 (61.0)	207 (71.4)	Ref.	
Under 3000 ksh per month	16 (39.0)	83 (28.6)	1.596	0.811 – 3.142
**Number of household member**				
Less than 6 persons	22 (53.7)	138 (47.6)	Ref.	
More than 7 persons	19 (46.3)	152 (52.4)	1.275	0.662 – 2.457
**Number of children under five**				
Less than 4 children	22 (53.7)	115 (39.7)	Ref.	
More than 5 children	19 (46.3)	175 (60.3)	1.762	0.913 – 3.400
**Birth place**				
at health facility	15 (36.6)	88 (31.8)	Ref.	
at own house / other	26 (63.4)	189 (68.2)	1.239	0.625 – 2.456
**Birth difficulty**				
Birth difficulty	34 (89.5)	244 (91.0)	Ref.	
No difficulty	4 (10.5)	24 (9.0)	1.196	0.391 – 3.657
**Neonatal insults**				
Neonatal insults	4 (10.5)	12 (4.5)	Ref.	
No insults	34 (89.5)	256 (95.5)	2.510	0.766 – 8.224
**Postnatal care**				
Less than 2 times	2 (4.9)	9 (3.1)	Ref.	
More than 3 times	39 (95.1)	281 (96.9)	0.625	0.130 – 2.997
**Full immunization**				
Full immunization	31 (75.6)	240 (89.2)	Ref.	
Not full	10 (24.4)	29 (10.8)	2.670^*^	1.187 – 6.003

^a^ Crude odds ratio.

^*^ p < 0.05.

^**^ p < 0.01.

^***^ p < 0.001.

**Table 4:** Multivariate analysis of risk factors for Neurological Impairment

**Risk factors**	**Adjusted Odds ratios**	**95% CI**	**P value**
**Maternal age**			
< 32 years old	Ref.		
≥ 32 years old	2.312	1.05 – 5.07	0.036
**Own children**			
< 5 children	Ref.		
≥ 5 children	2.85	1.25 – 6.49	0.013
**Income**			
More than 3001ksh per month	Ref.		
Under 3000ksh per month	2.79	1.28 – 6.08	0.010
**Tetanus shot**			
Tetanus shot in antenatal care	Ref.		
Non-tetanus shot in antenatal care	5.17	1.56 – 17.14	0.007

## Discussion

The prevalence of NI in the present study was 29/1000 and the most common impairment was cognitive (24/1000), followed by physical impairment (5/1000). Other comparable studies using TQQ were conducted in Kenya [12,34], India [26], South Africa [35], Ghana [36], Saudi Arabia [14], Jamaica [37], Bangladesh [24] and Pakistan [38]. In addition, TQQ cluster survey data was reported from 18 countries in the third round of UNICEF’s Multiple Indicator Cluster Survey 2005–2006 (MICS3) [9]. Within studies which assessed the validity of TQQ, the prevalence of NI ranged from 16 to 61 per 1000 and cognitive impairment was the most or second most common impairment. In a study in Kilifi, the prevalence of NI was 61/1000 and the most common impairment was epilepsy with prevalence 41/1000, followed by cognitive (31/1000), hearing (14/1000), motor (5/1000) and vision (2/1000) impairments. The prevalence of cognitive impairment in the present study was analogous to that in the Kilifi study [12]. However, the prevalence of cognitive impairment in other previous studies varies widely. The differences may be explained by the differences of definition of cognition and of age group, as children with severe disabilities will frequently die in infancy or early childhood. Moreover, the use of different assessment tools for cognitive impairment may considerably influence the prevalence of cognitive impairment. It is important to develop standardized assessment tools and definitions of cognitive impairment. However, the absence of standardized tools and lack of psychologists in our situation forced us to use a trained assessor and adapt the assessments for cognitive impairment that were culturally modified.

The prevalence of epilepsy and hearing impairment in this research was noticeably lower than in the research in Kilifi, Kenya [12]. It is well established that central nervous system (CNS) infection such as malaria and tuberculosis can lead to epilepsy [39,40] and the research site was one of the high malaria prevalence areas in Kenya. However, the prevalence of epilepsy was low in this study, compared with Kilifi study. It is estimated that the child who have epilepsy might be easy to die [41], because of stigma from community member, poor treatment and poor care from caregivers [41,42]. The other possibility is that the method used to diagnose epilepsy. In this study, caregiver recall was used to assess epilepsy. However, in Kilifi study, a interview by clinical officer and electroencephalogram were used to diagnose epilepsy, so that more minor epilepsy might have been found. The prevalence of hearing impairment (0.8/1000) in the present study was considerably lower than that (14/1000) in the Kilifi study [12]. Schooling rate to special school may influence the prevalence. There was one residential special school focusing on hearing impairment in Mbita district, and the phase two survey was conducted during schooling term. Some children with hearing impairment in Mbita district may have stayed in a special school.

The risk factors related to moderate/severe NI were low monthly income, having more children, maternal age and no maternal antenatal tetanus shot. Poverty is regarded as both a cause and consequence of disability [43]. Poverty and disability reinforce each other, contributing to increased vulnerability and exclusion [44]. It was clear that low monthly income was significantly related with impairment but it is not clear whether low monthly income is a cause or a result of disability based on the results in this study, because the research design was cross-sectional. Having more children was also related with moderate and severe neurological impairments. This finding was similar to the results reported in Saudi Arabia [14]. With an increased number of children in poor countries, quality of care for a child is likely to be worse because of competing demands on mothers, while time and resource available to provide for each child become more limited [45], so that severe disabilities are more likely to develop due to lack of care [46]. In addition, older maternal age was also related to have NI among children aged 6–9 years. As reported in other research, the older the woman, the greater the likelihood of miscarriage, stillborn or underweight baby, and likelihood of impairment also rises [47]. No maternal tetanus shot was another risk factor for NI. A study in Kenya reported that tetanus increased the risk of NI among survivors [48]. A mother who never received a tetanus shot was also less likely to go to antenatal care in this study. Moreover, antenatal care is related with child survival in terms of child fatal malnutrition [49] and neonatal death [50]. Therefore, even when the child survives, poor neonatal care [51], neonatal encephalopathy [11] and neonatal insults [12] increase the risk of NI among survivors.

Although neonatal insults was a risk factors of neurological impairment in other research [12,52], it was not significantly related in NI in this research. Further research on risk factors of NI would be better to ask more detail of neonatal insults.

There are four limitations in this research. There was a 1 year time-lag between phase one and phase two, because of ethical approval for physical assessment. Some children might have acquired impairments and died in the interim. However, since the age of first recognition of the impairment was mainly before 5 years old (86%) and the number of death was very few, the possibility of developing impairments or dying during the time-lag was supposed to be low. Moreover, around 100 households migrated during that time period and the households with CWDs might be likely to migrate. Hence, the years of staying in the community for households with CWDs were not different from Non-CWDs. Second, since our study design was cross-sectional, causal relationships between risk factors and impairments were not identified. A cohort study may be needed to illustrate the causal relationships. The third limitation was lack of a gold standard and standardized tools to assess cognitive impairment. The assessment for cognition in this research was an adapted and established cross-cultural assessment and definition of cognitive impairment were similar to the study in Kilifi. However, standardized tools and a gold standard for cognitive impairment are needed. Forth, since some CWDs might have stayed in special school during this research, there is a possibility that the prevalence of NI was underestimated.

## Conclusion

Poverty, a greater number of children, older maternal age and no maternal tetanus shot were risk factors of NI in the present study. Although further research is necessary, antenatal care services including a tetanus shot and education on how to take care of neonatal children are important to prevent neurological impairment based on the findings in this study. In addition, more attention to prevent NI should be put the family with poverty and a greater number of children.

## Competing interests

I declare that I have no competing interests.

## Authors’ contribution

YK was principal investigator for this research. SK conducted screening survey as phase one and supported to develop research design. MK coordinated some activities in Kenya and assisted to revise the structured questionnaire used in this research to match to Kenyan situation. SH also contributed a great deal in data collection and data analysis. All authors read and approved the final manuscript.

## Licence for publication

"The Corresponding Author has the right to grant on behalf of all authors and does grant on behalf of all authors, an exclusive licence (or non exclusive for government employees) on a worldwide basis to the BMJ Publishing Group Ltd and its Licensees to permit this article (if accepted) to be published in JECH editions and any other BMJPGL products to exploit all subsidiary rights, as set out in our licence ( http://group.bmj.com/products/journals/instructions-for-authors/licence-forms/)."

## Pre-publication history

The pre-publication history for this paper can be accessed here:

http://www.biomedcentral.com/1471-2431/12/186/prepub
